# Moving from information and collaboration to action: report from the 3rd International Dog Health Workshop, Paris in April 2017

**DOI:** 10.1186/s40575-017-0054-4

**Published:** 2017-12-07

**Authors:** Dan G. O’Neill, Sylvia F. A. Keijser, Åke Hedhammar, Caroline Kisko, Gregoire Leroy, Aimée Llewellyn-Zaidi, Sofia Malm, Patricia N. Olson, Rowena M. A. Packer, Jean Francois Rousselot, Ian J. Seath, Jason W. Stull, Brenda N. Bonnett

**Affiliations:** 1 Pathobiology and Population Science, The Royal Veterinary College, Hawkshead Lane, North Mymms, Hatfield, Herts AL9 7TA UK; 2 Expertise Centre Genetics of Companion Animals, Department of Clinical Sciences of Companion Animals, Faculty of Veterinary Medicine, Utrecht University, Utrecht, The Netherlands; 3 Department of Clinical Sciences, Swedish University of Agricultural Sciences, Uppsala, Sweden; 4 The Kennel Club, Clarges Street, London, W1J 8AB UK; 5 AgroParisTech, UMR 1313 Génétique Animale et Biologie Intégrative, F-75231 Paris, France; 6 INRA, UMR 1313 Génétique Animale et Biologie Intégrative, F-78352 Jouy-en-Josas, France; 7 International Partnership for Dogs, c/o Svenska Kennelklubben, Rinkebysvängen 70, SE-163 85 Spånga, Sweden; 8 Svenska Kennelklubben, Box 771, SE-19127 Sollentuna, Sweden; 9 Affiliate Faculty, Colorado State University, Fort Collins, CO 80523 USA; 10 Clinical Science and Services, The Royal Veterinary College, Hawkshead Lane, North Mymms, Hatfield, Herts AL9 7TA UK; 11 Clinique vétérinaire du Clos des Camélias, 72 Bd Charles de Gaulle, F 92701 Colombes, France; 12 Chairman Dachshund Breed Council UK, London, UK; 13 Department of Veterinary Preventive Medicine, College of Veterinary Medicine, The Ohio State University, Columbus, OH 43210 USA; 14 International Partnership for Dogs, Georgian Bluffs, Ontario Canada

**Keywords:** Welfare, IPFD, DogWellNet, Exaggeration, Extreme, Behavior, Genetics, Big data, VetCompass™, PETscan

## Abstract

**Background:**

Breed-related health problems in dogs have received increased focus over the last decade. Responsibility for causing and/or solving these problems has been variously directed towards dog breeders and kennel clubs, the veterinary profession, welfare scientists, owners, regulators, insurance companies and the media. In reality, all these stakeholders are likely to share some responsibility and optimal progress on resolving these challenges requires all key stakeholders to work together. The International Partnership for Dogs (IPFD), together with an alternating host organization, holds biennial meetings called the International Dog Health Workshops (IDHW). The Société Centrale Canine (French Kennel Club) hosted the 3rd IDHW, in Paris, in April, 2017. These meetings bring together a wide range of stakeholders in dog health, science and welfare to improve international sharing of information and resources, to provide a forum for ongoing collaboration, and to identify specific needs and actions to improve health, well-being and welfare in dogs.

**Results:**

The workshop included 140 participants from 23 countries and was structured around six important issues facing those who work to improve dog health. These included individualized breed-specific strategies for health and breeding, extreme conformations, education and communication in relation to antimicrobial resistance, behavior and welfare, genetic testing and population-based evidence. A number of exciting actions were agreed during the meeting. These included setting up working groups to create tools to help breed clubs accelerate the implementation of breed-health strategies, review aspects of extreme conformation and share useful information on behavior. The meeting also heralded the development of an online resource of relevant information describing quality measures for DNA testing. A demand for more and better data and evidence was a recurring message stressed across all themes.

**Conclusions:**

The meeting confirmed the benefits from inclusion of a diverse range of stakeholders who all play relevant and collaborative parts to improve future canine health. Firm actions were set for progress towards improving breed-related welfare. The next international workshop will be in the UK in 2019 and will be organized by the UK Kennel Club.

## Plain English summary

Breed-related health problems in dogs have received increased attention in the media and veterinary literature over the last decade. Responsibility for causing and/or solving these problems has been variously laid at the door of dog breeders and kennel clubs, the veterinary profession, welfare scientists, owners, regulators and the media. In reality, all these stakeholders are likely to share some responsibility and optimal progress on resolving these challenges will be achieved only if key stakeholders can coordinate and work together. This article presents a summary of the structure, goals and outcomes of a large meeting held in Paris in April 2017 that aimed to develop an action plan on breed-related health in dogs.

The 3rd International Dog Health Workshop provided a forum for formal and informal discussions between all relevant groups where key issues were identified and defined, and plans were agreed for effective actions to address them. In total, 140 participants from 23 countries attended and the workshop was structured around six important issues facing those who work to improve dog health. These included individualized breed-specific strategies for health and breeding, extreme conformations, education and communication in relation to antimicrobial resistance, behavior, genetic testing and population-based evidence. Short plenary presentations from international experts were followed by two days of breakout sessions that were designed to maximize communication and networking while at the same time clustering recognized experts to encourage original thinking and solutions.

The meeting confirmed the benefits from inclusion of a diverse range of stakeholders who all play relevant and collaborative parts to improve future canine health. A number of exciting actions were agreed during the meeting. These included setting up working groups to create tools to help breed clubs accelerate the implementation of breed-health strategies, review aspects of extreme conformation and share useful information on behavior. The meeting also heralded the development of an online resource of relevant information describing quality measures for DNA testing. A demand for more and better data and evidence was a recurring message stressed across all themes. The next international workshop will be held in the UK in 2019 and will be organized by the UK Kennel Club.

## Background

Breed-related health problems in dogs, especially inherited diseases in pedigreed dogs, have received increased attention in the media and veterinary literature over the last decade, and this has been followed inevitably by a public blame game [1]. Some place the responsibility for breed-related health problems firmly on dog breeders and kennel clubs by focussing on ill-advised selective-breeding decisions and lack of proactive measures for dog health [2]. Other authors have suggested that the veterinary profession could have been more proactive [3, 4], while yet other studies have addressed the role of consumer attitudes and actions [5, 6]. In reality, all these stakeholders, as well as others such as the media and celebrities who popularise certain breeds [7, 8], are likely to share some responsibility because each plays important but differing roles in promulgating various aspects of this complex issue of breed-related health problems in dogs. Efforts to understand and address health and welfare problems in dogs are complicated by issues around the sourcing of puppies. In many countries, the majority of apparently purebred dogs are thought to come from commercial breeders who are not registered with relevant kennel clubs and therefore may fall outside the normal influences, controls and regulations of such bodies (www.humanesociety.org). Clearly, this all leads to a very complex situation and optimal progress on resolving these challenges will be achieved only if key stakeholders can coordinate and work together to embrace positive and evidence-based change. A critical element required for such progression is the provision of a forum for formal and informal discussions between all relevant groups where key issues can be identified and defined, and plans can be agreed for effective actions to address them.

There are undoubtedly many important issues facing those who work to improve dog health. In this report, we focus on six in particular. Although some over-arching concepts may apply across all dogs worldwide, individualized breed-specific strategies for health and breeding are needed and may vary by country [9, 10]. Complex conditions, such as those associated with brachycephaly and other extreme conformations, negatively impact not only the health but also the welfare of individual dogs [11, 12]. The intricacies facing stewards of well-being in dogs including kennel clubs, breeders, veterinarians, scientists and regulators are such that collaborative, international and multi-stakeholder efforts on education and communication are needed across many topics including in relation to antimicrobial resistance [4, 13]. In order to breed healthier dogs, many other aspects of canine health need to be considered, as not all challenges are traceable solely to genetic influences; for example, disease, behavior, and welfare also interact to influence dog health [9]. Great advances in the study of genetic disease have led to a growing plethora of genetic tests but the complexity of optimal usage of these tests has also caused breeders, kennel clubs and breeding advisors to struggle as they try to reduce the burden of inherited diseases in the dog population [10, 14]. And finally, the true burden of disease within individual breeds, as well as across national populations of dogs, is poorly understood and there is little reliable population-based and generalizable evidence to quantify the prevalence of various conditions or their comorbidities [15].

In June 2012, the first International Dog Health Workshop (1st IDHW) [16] was organized by the Swedish Kennel Club and held in Stockholm, Sweden as a satellite meeting to the 6th International Conference on Advances in Canine and Feline Genetics and Genomics. The 1st IDHW brought together representatives from many of the groups that share a responsibility for dog health. Numerous recommendations came from the workshop, including that an international platform for collaboration among stakeholders in dog health and welfare should be developed (i.e., a suggested prototype that later spawned the International Partnership for Dogs (IPFD) and DogWellNet.com) [17]. Another key recommendation was that (standardized) procedures for validation of both DNA-tests and testing laboratories should be defined and communicated, along with recommendations for proper use of genetic testing in different populations (which has led, eventually, to the Harmonization for Genetic Testing in Dogs (HGTD) initiative, see below*).* Developments in genetic testing are progressing faster than the general public may realize so it is important to parallel reliable public-facing communication systems to ensure optimal uptake of these novel methods.

The 2nd International Dog Health Workshop (2nd IDHW) in Dortmund, Germany in February 2015 was coordinated by the IPFD and the German Kennel Club (VDH) [18]. This meeting marked the launch of DogWellNet.com [17], the internet platform of the IPFD which was registered as a non-profit organization in August 2014. The IPFD was initiated by several national Kennel Clubs (Sweden, Finland, Germany, France, Norway, the UK and the USA) and other stakeholders in dog health including The Orthopedic Foundation for Animals, USA [19] and the Agria Pet Insurance-SKC Fund, Sweden. The Fédération Cynologique Internationale (FCI) [20] represents 91 national kennel clubs and is an Initiating Patron and Member. The Irish KC [21] is a partner; and, current collaborating partners also include VetCompass™ (UK) [22], the Australian Shepherd Health and Genetics Institute (ASHGI0 [23] as well as this journal (Canine Genetics and Epidemiology, CGE). More recent Corporate Partners include Mars Veterinary and Royal Canin while additional collaborators and sponsors are being sought from all stakeholder groups. The IPFD’s mission is to facilitate collaboration and sharing of resources to enhance the health, well-being and welfare of pedigreed dogs and all dogs worldwide.

In April 21–23, 2017 the IPFD 3rd International Dog Health Workshop (3rd IDHW) [24] was hosted by the Société Centrale Canine (French Kennel Club) in Paris, France. Major sponsors were Agria Pet Insurance (Sweden, UK, France) and Royal Canin. The objective of this article is to present a summary of the structure, goals and outcomes of this meeting that can inform and engage stakeholders and act as a blueprint for progress assessment at the planned 4th IDHW in 2019 in the UK.

## Methods

### Meeting format

The 3rd IDHW followed a similar format to the previous meetings. Organized along a working and networking framework, the IDHWs are designed to identify and prioritize issues and challenges in breeding, health and welfare of dogs, to encourage dialogue across stakeholder groups, to promote international collaboration and action, and to define and address common goals. In total, 140 participants from 23 countries attended the 3rd IDHW and comprised decision-leaders from most major stakeholder groups in dog health and welfare. The attendees were diverse and included breeders, members of breed club health committees, kennel clubs, breeding advisors, veterinarians, educators, researchers, geneticists, behavioral specialists, regulators, welfare organizations, industry, media, health campaigners, show judges and dog owners.

The meeting was formatted around the 6 key themes outlined above as issues that regularly feature as discussion points in relation to breed-related health in dogs (Table 1). Short plenary presentations from international experts on the morning of the first day were followed by breakout sessions for each theme over the two days that were interspersed with two sharing sessions in plenum. The format was designed to maximize communication and networking while at the same time clustering recognized experts within theme hubs to encourage original thinking and solutions.

**Table 1:** Six overall themes for the 3rd International Dog Health Workshop in 2017 in Paris, France

Theme	Session leader(s) (number of participants)
Breed-specific health strategies: needs and opportunities; innovations, nationally and internationally.	Helena Skarp, Sweden; Ian Seath, UK; Gregoire Leroy, France. (34)
Exaggerations and extremes in dog conformation: health, welfare and breeding considerations; latest national and international efforts.	Åke Hedhammar, Sweden; Rowena Packer, UK; Kristen Prestrud, Norway (27)
Education and communication: how can international collaboration improve education and communication within and across stakeholder groups [especially between veterinarians and breeders]; using the example of antimicrobial resistance.	Gilles Chaudieu, France; Jason Stull, USA (13)
Behavior and welfare: how can we better integrate actions to address issues in welfare, behavior and health in breeding and raising dogs?	Nathalie Marlois, France; Patricia Olson, USA; Caroline Kisko, UK (15)
IPFD Harmonization of Genetic Testing for Dogs: an international, multi-stakeholder initiative to address selection, evaluation and application of genetic testing.	Aimee Llewellyn-Zaidi, USA; Brenda Bonnett, Canada (34)
Show me the numbers: integrating information from various sources for prevalence, risks and other population-level information; latest national and international strategies to collect data and disseminate information.	Dan O’Neill, UK; Sylvia Keijser, The Netherlands; Sofia Malm, Sweden (14)

From the outset, it was emphasized to all delegates that IPFD and the 3rd IDHW aimed to provide the forum and structure to support collegiate progress in dog health but that it was neither the mandate nor the intent of IPFD to directly produce regulations or directives. The participants were provided with information relevant to their specific themes in advance of the conference in order to focus activities both during and after the meeting. Possible outcomes suggested as desirable from the themes included sharing of existing information, templates, and tools; identification and prioritization of key actions to support breed-related health; development of collaborative strategies and community building. The strapline for the 3rd IDHW was ‘from information and collaboration to action’ and therefore, a priori, the meeting aimed to go beyond mere discussion to generate meaningful outcomes. Pre- and post-meeting resources and material for the 3rd IDHW are available on DogWellNet.com [17]. This paper summarizes the discussions, recommendations and actions identified and committed to by participants during the 3rd IDHW.

## Results

### Work themes and outcomes

As described above, the meeting was structured around 6 key themes that were identified in advance as offering substantial opportunity for action to improve breed-related health in dogs (Table 1). Each theme is described below with information provided on the discussions that took place and any actions proposed by participants.

#### Breed-specific health strategies

Breeding advisors, breed clubs and individual breeders frequently struggle with two main issues. First, how to define and understand the ‘big picture’ for their breed in terms of disease, genetics, population numbers, breeding and general management. Second, how to process all the complex inputs that affect the health and welfare of their dogs. Without access to full information, adequate evidence or effective tools to define the big picture, stakeholders tend to view challenges more narrowly and in the shorter term. This may mean that they end up running after the DNA ‘test of the month’ or imposing knee-jerk reactions to media storms that may lead to breeding strategies that change again as soon as the executive of the breed club changes. Optimal and selected approaches to managing health and disease at a breed level also vary widely across countries, kennel clubs, breed clubs and breeds. It is therefore important to build on experiences from these different countries and groups, in order to facilitate exchange and collaboration, harmonize health assessment and screening programmes, and suggest optimal strategies and health strategies for use at the breed level. This was the background to the session on breed-specific health strategies.

The participants in the session were truly multi- and inter-disciplinary, and included geneticists, veterinarians, epidemiologists as well as breeders, owners and dog-health campaigners. With 34 participants, this was the most popular stream at the workshop. Ian Seath (UK), Chairman of the UK Dachshund Breed Council [25], shared his experience based on the approach taken by his breed council and stressed the importance of applying accepted business management elements including leadership, planning, engagement and improvement.

The group agreed that effective and sustainable implementation of health strategies requires innovative solutions to many different challenges. Provision of sufficient and reliable information was agreed as critical, for both situational assessment as well as day-to-day screening of dogs. On the one hand, a diversity of survey templates for breed health assessments have been developed and are available for individual breeds [26]. On the other, veterinary screening programmes and diagnosis-based research requires harmonization across breeds for effective application in health programmes, especially at an international scale. Considering the design of health strategies, the group decided that it was important to identify and balance the major issues for each individual breed and give guidelines on how priorities could be determined for each [4], while still allowing breeders discretion to make their own decisions within an overall framework of requirements and recommendations. The group agreed that it would be useful to develop a model to evaluate generic breed problem categories (e.g. inherited disorders with DNA test available, multi-factorial conditions with existing screening programmes) in order to define breeding strategy solutions (e.g. breeding recommendations based on DNA tests adapted to disorder prevalence, development and use of estimated breeding values (EBV)). Importantly, the group also concurred on the deleterious consequences of inappropriately removing dogs from the breeding gene pool when breeders failed to understand the conflicting influences and effects that may arise from disease control strategies versus a need for genetic diversity.

The group considered that achieving compliance by breeders and owners to recommended or required screening and breeding guidelines was a challenge for breed-based health strategies. Imposition of mandatory screening programmes and open registries of test results as a prerequisite for kennel club registration could result in breeders choosing to breed non-registered dogs instead. However, lack of adherence to programmes and incomplete data pose significant barriers to achieving health improvement. This underlined the importance of education and communication between the different stakeholders (including breeders, owners, veterinarians, geneticists and judges) in the design and implementation of effective health strategies.

The group discussed their diversity of experiences across countries in relation to breed-specific health strategies and access to their local resources and tools that could be shared more widely. The use of the DogWellNet.com platform website as a repository for such resources was recommended. The general conclusion was that there is no “one size fits all” solution for developing breed-specific health strategies and that the most effective interventions would be adapted according to the specific context of each breed [27]. The impact of national cultures on successful approaches can be significant. For example, the Nordic countries enjoy a culture of regulation and compliance from breeders and have advanced breed-specific strategies in place. However, a similar regulatory approach would risk driving breeders away from their kennel club’s sphere of influence in other locations (e.g. the Benelux and Southern Europe regions).

The group felt that a more holistic approach to breeding was needed, with greater focus on population-specific situations and reduced emphasis on breeding decisions based solely on single diseases and DNA tests. To that extent, the group considered that it was inadvisable to conduct health strategies within individual breeds focused on single diseases independently from a more broadly focused breeding strategy. The participants agreed to set up a working group, led by Jerrold Bell and including 12 other participants from the workshop, to take forward the ideas discussed and to create a set of resources and tools that could help breed clubs to accelerate the creation and more importantly, the implementation, of strategies that benefit their dogs. Subgroups from this working group will also work on the development of breeding strategies for specific breeds, such as the Bernese Mountain Dog and Dachshund.

#### Exaggerations and extremes in dog conformation

As the popularity of small-sized flat-faced breeds continues to increase around the world, the health and welfare of brachycephalic breeds has become an increased priority issue. Rowena Packer (UK) outlined current understanding of health consequences from extreme brachycephaly in her plenary presentation and described mounting evidence of breathing, thermoregulation, eye, skin, spinal and birthing problems associated with this phenotype [28–32]. In consequence, this theme elected to focus exclusively on brachycephalic health, with specific emphasis on breathing problems (brachycephalic obstructive airway syndrome; BOAS) that were considered the most severe welfare concern in brachycephalic breeds [33, 34]. However, key points of the discussion also relate more generally to other issues of exaggerations and extremes.

Discussions across the 27 participants covered both current efforts, such as the formation of brachycephalic working groups in the UK [35] and by the Nordic Kennel Union [36], whilst also debating alternative future strategies. Although kennel clubs have developed initiatives to improve brachycephalic health (e.g. ‘Breed Watch’ in the UK [37], ‘Breed Specific Instructions’ in the Nordic region [38]), significant challenges remain. It is increasingly clear that the brachycephalic issue is largely a ‘human’ problem, with change hindered by frequent ‘blindness’ to the health problems in these breeds, and ‘normalization’ of their health issues [39, 40].

The group formulated 5 goals to improve brachycephalic health:

1. Kennel clubs and the FCI should further educate breeders and judges on brachycephalic health and police those who promote unhealthy practices; encourage/enforce fitness tests [41, 42] prior to breeding/showing, and review breed standards to remove features detrimental to health and increase their objectivity.
2. Show judges should be well-educated on the detrimental consequences of extreme conformation; interpret breed standards with canine health in mind; and only award prizes to less extreme dogs that are free of signs of ill-health.
3. Breeders should choose less-exaggerated breeding stock that have undergone appropriate health testing for breeding.
4. Puppy buyers should have enough knowledge to make informed choices, should not focus solely on looks and should demand increased health testing and reduced exaggeration.
5. Veterinarians should be actively involved in breed health, via e.g. breed health testing; education of puppy-buyers via pre-purchase visits; and participation in data collection (e.g. reporting of conformation-altering surgery and caesarean sections) and sharing clinical data with national epidemiological research and governance programmes.

To achieve these goals, sub-groups were created, who will work to:

1. Document ongoing international projects on brachycephalic health to promote collaboration and share best practices.
2. Compare current methods to measure exercise tolerance with a view to validation and harmonization.
3. Quantify brachycephaly-related disorders in registered and non-registered populations.
4. Identify phenotypic and genetic variation *within* breeds to evaluate whether this variation can be utilized as an alternative to outcrossing e.g. unregistered dogs and breed variants.
5. Review breed standards to highlight points that encourage exaggeration or allow misinterpretation.
6. Evaluate the ways in which human behavior can be changed; including judges, breeders and puppy-buyers.
7. Influence media portrayal of brachycephalic breeds to move from promotion of extreme breeds in mainstream advertising to communication of educational messages.

The working groups are committed to these actions, and joint coordinator Kristin Prestrud has presented these plans at the WSAVA/FECAVA congress 2017 [43].

#### Education and communication of antimicrobial resistance

The emergence and expansion of antimicrobial resistance (AMR) has been widely documented and challenges current antimicrobial therapy protocols. It has increased human and veterinary treatment costs and patient morbidity and mortality [44, 45]. AMR is geographically widespread and can be transmitted between humans and animals [46, 47]. AMR remains a challenge in veterinary medicine with limited and differing guidelines across countries that results in fragmented communication and education approaches.

The AMR theme subgroup reviewed selected materials covering national AMR guidelines [48–51], antimicrobial prescribing pressure in healthcare [52], and AMR transfer between people and companion animals [53] prior to the meeting. The conference plenary presentation from Jason Stull (US) further explored these topics and highlighted issues such as unnecessary/inappropriate antimicrobial prescribing in medicine, lack of studies addressing usage in breeding dogs, and stressing the importance of targeting behavioral change in antimicrobial use at multiple levels (i.e., intra-personal, inter-personal, community, institutional) [54–56].

The subgroup included 13 participants representing five countries from sectors including academia, veterinary medical associations, private practice, pet insurance, kennel clubs and foundations. An initial presentation reviewed actions taken in France to address AMR in companion animals, including development of surveillance collaboration with veterinary practitioners and laboratories (RESAPATH) [55], recent policy and law to reduce usage of critically important antimicrobials, guidelines to promote prudent antimicrobial use, and training and campaigns to create awareness. Following these efforts, a 20% reduction in antimicrobial use in animals was observed (2011 to 2015; estimated to be 10% reduction in dogs and cats) [56]. Other countries have employed similar approaches with comparably successful outcomes [57].

Challenges discussed to replicating the French model in other countries included limited stakeholder buy-in, strong lobbying groups, resistance to top-down approaches, and varying backgrounds of breeding groups across countries. Additional challenges included sustaining and enforcing prescribing requirements and antimicrobial use reporting. Lack of published antimicrobial usage and AMR data in breeding dogs and limited prudent-use guidelines for breeders and veterinarians were considered major limitations. Participants agreed that veterinarians should work collaboratively with breeders to effect change and that a multi-national educational approach aimed at breeders was needed to unify groups and drive positive change.

The group identified four main future priorities to address AMR in dogs:

1. Create a global AMR network comprising key stakeholder groups across countries including IPFD, kennel and breed clubs, veterinary medical associations, and industry.
2. The global AMR network would develop and promote (if not already in-place) antimicrobial use guidelines for breeders and veterinarians aimed at general healthcare and conditions specific to breeding (e.g., use surrounding breeding and whelping) and dog shows (e.g., gastrointestinal signs associated with stress).
3. Identify and develop funding initiatives to support research and surveillance efforts with breeding groups and provide data (antimicrobial use, resistance and perceptions) to support and provide feedback on established guidelines. Relevant studies might include literature review and data collection specific to AMR, breeding, and antimicrobial use practices; studies establishing normal and antimicrobial-induced alterations to relevant microbiomes (e.g., vaginal).
4. Development of certificate and learning modules for breeders and veterinarians in order to provide education and communicate developed guidelines. Module materials would include information on negative outcomes from imprudent antimicrobial use and alternative approaches to antimicrobials. The modules would encourage the use of storytelling to personalize the issue and target intra-personal, inter-personal and community pressures to alter behaviors.

Given international differences in culture and infrastructure, it is perhaps unsurprising that there is currently a fragmented approach to addressing AMR in dogs across country/region and stakeholder groups. The discussions of this multi-stakeholder international group highlighted the limited information currently published on this topic in breeding dogs and that a unified approach is required to capitalize on current successes and resources. This conference and resulting working groups are an excellent step toward these concordant efforts.

#### Behavior and welfare

Socialization of puppies at appropriate ages is considered critical for optimal behavioral development of dogs to facilitate their life as pets within human homes. Dogs with appropriate behavioral responses are more likely to remain with owners or adopters, thereby strengthening the human-animal bond and promoting animal welfare and human well-being. Conversely, dogs that display undesirable behaviors may have compromised welfare driven by their underlying emotional motivations for the behavior (e.g. anxiety) or from how owners/adopters might seek to achieve resolution (e.g. aversive techniques, relinquishment) [58–60].

A thought-provoking plenary talk from Paula Boyden (UK) entitled *The intersection of welfare and behaviour in dogs and relation to health and breeding* set the tone for the theme by focusing on socialization in puppies. Some complex interactions across this topic were highlighted including selection for physical features that may limit expression of normal behavioral communications with dogs and people, and early life experiences that impact later health and welfare and influence human-animal interactions. Examples of puppy programs that support development of positive health behaviors were also described.

The theme included 15 participants with diverse backgrounds from eight countries (France, Sweden, Switzerland, Finland, Germany, Ireland, UK, and USA). The group explored knowledge and beliefs around several aspects of puppy socialization that relate to later behaviors and animal welfare. Topics discussed included the critical sensitive period for socializing, evidence for outcomes with different socialization methods, potential breed differences, gaps in knowledge, access to international literature on the subject, existing programs that might be replicated/tested, correlations between puppy testing and future outcomes of behavior, educational needs for new owners, and educational needs for breeders and other stakeholders.

This group particularly focused on setting goals and refining specific actions to achieve these goals. Six key goals were developed during the workshop:

1. Behavioral consideration should form part of routine pre-breeding decision-making by contributing to breeding choices (e.g. temperament of bitch and sire). Good management should aim to minimize stress throughout pregnancy.
2. Improved behavioral education of breeders (novice, professional, commercial), veterinarians, veterinary students, allied health professionals, novice and experienced owners and handlers.
3. Address issues that may adversely affect ideal socialization including sourcing issues such as importation, puppy mills/farms, pet stores.
4. Develop simple and powerful public messages that promote the benefits of purchasing an appropriately socialized puppy.
5. Determine which (if any) excellent socialization programmes already exist, and replicate widely.
6. Consider that individual puppies may require adapted socialization protocols.

The participants prioritized 5 action items to be addressed by members of the group over the following 24 months.

1. Prepare public messages that will promote the acquisition of well-socialized puppies.
2. Conduct a comprehensive, international literature review to identify evidence-based socialization/puppy testing methods.
3. Following this literature review, identify research gaps whereby academic centers might generate topics for future scientific studies of socialization methods (e.g. longitudinal/prospective studies).
4. Identify previously unpublished but useful data that might be analyzed and published to increase the body of evidence on socialization.
5. Survey national kennel clubs for socialization materials/resources that could be validated and replicated internationally.

Throughout the workshop, the information and experiences shared by participants were highly instructive and led to shared goals for international collaboration. The group agreed that puppy socialization has many important requirements, from providing excellent prenatal care, to minimizing stress throughout pregnancy and minimizing fear with proper housing, addressing critical times for introducing puppies to novel environments/people, and determining the evidence/outcomes for various methods utilized. While proper socialization should not be considered the only criterion for producing healthy puppies, it was deemed necessary for developing a dog with good behavior and a better chance for a good life.

#### IPFD harmonization of genetic testing for dogs

Increasing demand for genetic testing has led to a boom in for-profit and non-profit, commercial and academic genetic test providers (GTPs) and available tests [61]. Defining “good quality” GTPs and DNA testing, in the current absence of independent regulation, is almost impossible for dog owners, veterinary scientists, and breed/kennel clubs [62].

In parallel with an increase in breeding policies incorporating genetic testing [63], there are no standards, regulations, or quality control metrics for GTPs providing DNA testing in veterinary medicine. Along with anecdotal experiences of poor GTPs, this brings genetic testing in dogs broadly into disrepute, and disincentivizes conscientious GTPs to maintain high standards. Even in human testing, serious questions are raised about the regulation of medical testing [64].

In response to, and building on discussions at the 1st IDHW and 2nd IDHW, the IPFD is overseeing the development of an online resource of relevant information from GTPs describing quality measures (QMs) for DNA testing. Further development into 2018 will include platforms for expert reviews of tests; coordinating a proficiency testing scheme, and genetic advice and education. The model depends on GTPs and multi-stakeholders participating voluntarily. An open-access prototype was developed using data provided by GTPs indicating a spectrum of initial QMs, from international accreditations to customer care. This centralized resource aims to aid kennel/breed clubs, breeding advisors and owners to make better informed decisions on GTPs and testing.

The 34 theme participants included representatives from GTPs, geneticists and researchers, kennel clubs/registration bodies, and owners/breeders. In preparation for the 3rd IDHW, theme participants were provided with a reading list including the prototype description, and recommended websites of similar systems in human/non-companion animal testing (www.dogwellnet.com, www.eurogentest.org, www.orpha.net, www.icar.org, www.acmg.net). Objectives for the workshop were to encourage stakeholder engagement with the project and to identify experts/ participants for future development of the platform.

Following a plenary presentation from Aimée Llewellyn-Zaidi (US), the theme discussed issues including the independent evaluation of GTPs, individual DNA tests, and genetic advice. The group accepted that most genetics experts affiliate with at least one GTP and therefore may not be truly unbiased. To address this, the IPFD was identified as an independent organization capable of leading a strategy of balanced and collaborative compilation of quality information on GTPs.

The group felt that building a definitive list of current QMs and GTPs was paramount. An agreed action was to host this list on DogWellNet.com (expected early 2018). Concerns were raised on standardizing QMs across international boundaries and laboratory types (i.e. commercial vs. primarily research laboratories). The result was to form a working group of multi-stakeholders and laboratories to be hosted on a DogWellNet.com forum.

Future priorities included development of a proficiency testing scheme and collation of resources for genetic advice. This lead to forming working groups to address evaluation of genetic testing, advice, sustainability, and proficiency testing. Leaders for each working group are experts in relevant fields, and a balance across stakeholders was determined. External experts would be sought where relevant.

The group considered that the lack of accreditation and standardization across DNA testing is putting the health of dogs at risk. Without adequate guidelines, or external validation, consumers risk making detrimental breeding decisions based on irregular results, or fraudulent activities. Without consumer confidence in DNA testing, GTPs and researchers will struggle, and preventable inherited diseases will continue. The group agreed that the Harmonization of DNA testing for Dogs project, is a major step towards engaging with GTPs, and experts, to improve use of DNA testing.

#### Show me the numbers: Integrating information from various sources for prevalence, risks and other population-level information

Data-deficiencies are widely acknowledged to constrain improvement in companion animal health [15]. A demand for more and better data was identified across each of the other five themes with a recurring message that actions should ideally be based on good evidence wherever possible. The Numbers theme aimed to identify opportunities to increase the availability of data in order to improve dog health. With 14 participants from six countries, the Numbers group benefitted from inclusion of leading representatives from academia, animal insurance, kennel clubs, data analysis, laboratories and business, enabling discussion on a wide range of data topics.

Participants were provided with selected pre-meeting reading material covering data limitations and opportunities (e.g. [65–69]. A plenary talk from Sofia Malm (Sweden) discussed integration of information from various sources. The first breakout session stimulated debate on the epistemological nature of information as theme leaders, Dan O’Neill (UK) and Sylvia Keijser (Netherlands), directed participants to consider why specific types of health knowledge are often unknown or ignored [70]. All 14 participants contributed enthusiastically and openly during the two-day discussions which identified four main data areas:

1. Data access and the representativeness of data: The group discussed that true representativeness requires a national dog registry and should also include designer types and non-pedigreed purebred dogs [68]. Openness in data sharing was encouraged, but with some caution because of the complexity of such data and challenges to proper interpretation including the choice of appropriate control groups [71].
2. Multifaceted roles for veterinarians: Veterinarians hold key opportunities for generating and disseminating health data in collaboration with owners/breeders. Examples of successful veterinary data initiatives were cited including VetCompass™ in the UK and Australia [22, 72] and PETscan in the Netherlands [73].
3. Some key factors around data collection:
  - Cultural impact: each country has its own cultural incentives and potential sources of information that need to be considered for successful data collection.
  - Impact of funding: passive ignorance of alternative topics is risked when funding focuses on one area. For example, government funding focussed on dangerous dogs could lead to avoidance of welfare research.
  - Stewardship: the end-users and purposes of the data should be determined in advance to ensure optimal gains.
  - Dissemination: for real-world impact, data should affect the decisions and actions of stakeholders.
4. Prioritization of data needs:
  - Better demographic information was a core need.
  - Information on prevalence/incidence, risk factors, and geographic spread, as well as, genetic background to disorders and genetic structures of populations. Capturing trends on emerging diseases, for example, could then create predictive data.
  - Quality-of-life and end-of-life data capture was also considered very important. These data could predict breed longevity, and estimate summary measures of population health (SMPH) such as disability adjusted life years (DALY) and quality adjusted life years (QALY) [74]. The group considered that DALY and QALY data may be more relevant welfare indicators than longevity.

These four main data areas were also echoed from each of the other themes. Some additional numbers-based comments from the other themes included the value of longitudinal evaluation of breed health to assess the impact of programs and that data collection efforts (e.g. breed club health surveys, antibiotic use and AMR, behavioral assessments) need to be enhanced and coordinated. Additional actions to facilitate progress on all identified needs included publishing data results, for example in CGE; creating a meeting place for people who have data or questions regarding data on DogWellNet.com; and exploring funding for knowledge sharing and working together on an international level.

### Poster presentations

Attendees were offered the option to present a poster on topics of relevance to the themes of the 3rd IDHW. The poster presentation proved very popular and included 24 posters that represented research from breed clubs, scientists, students, veterinarians and breeders, and covered not only specific studies but also educational and breed-specific programs. The posters offered the authors the chance to present their institution or work in an efficient manner to a large audience while other attendees were easily able to identify useful connections and concepts that might offer future collaborative potential. Posters were not orally presented or judged in order to remove any competitive element; instead the aim was for breadth of topics, easy access and general benefit.

## Discussion

The 3rd IDHW was structured around 6 key themes. Attendees were allocated to their specific theme and stayed with this group for the duration of the meeting. In effect, each theme began in the weeks leading up to the meeting with the provision of open delegate lists and selected reading lists for each theme to encourage prior preparation and discussion. At the meeting, the tone for each theme was set by a plenary talk followed by a series of dedicated break-out sessions. To further increase productivity, each theme had at least two session leaders who had been involved for several months in its design and who provided an overview of possible discussion topics and a reference list on various work to focus the thoughts of participants. In addition, each theme was assigned a note-taker to ensure that all ideas and comments were formally recorded to assist with later dissemination. This strategy, together with efforts coordinated by the IPFD to identify and encourage the attendance of key decision leaders from the dog world, resulted in higher levels of active contribution from each individual compared with traditional conference formats that rely on mainly didactic lecture programmes and it fitted the aims of the meeting which were to generate new collaborations and actions. This structure could be recommended for future meetings that aim for high participant engagement.

The 3rd IDHW aimed a priori to move from information and collaboration to action. Although the precise outcomes could not be predicted in advance, the lists of actions agreed upon during the meeting suggest that this aim was largely achieved. Within each theme, participants determined their own specific priorities and challenges, and created their own lists of opportunities and needed actions. These reflected the goals of identifying priorities, gaps and actions, as well as building on international communication and collaboration. In many cases, firm plans were drawn up during the meeting to meet these actions. Working groups with specific tasks were identified and many plan to communicate through forum communities on DogWellNet.com. Each of these outcomes are hugely welcome for their own direct value but also because they are strong evidence of the willingness of the various stakeholders to share data and resources and to work as teams for the greater benefit of canine health. The greatest challenge will be to continue the positive momentum generated by the meeting into sustained action. At the time of publication, several groups from the conference remain very active. Another exciting development post-workshop was an IPFD veterinary student project which has assembled resources on the AMR topic (https://dogwellnet.com/content/hot-topics/antimicrobial-resistance-prudent-use-of-antibiotics/antimicrobial-resistance-resources-r488/).

The poster exhibition represented another effective communication strand from the meeting. The posters allowed participants an opportunity to share their work across the spectrum of attendees, to trigger a two-way dialogue on the work and to build new networks for the future. The presentation of activities and programs, in addition to research, allowed attendees to connect with others working on similar issues. This increased awareness of developments in canine health will underpin sustained collaborative efforts.

Diversity among the participants has been a noticeable feature of all IDHWs. The Workshops focused particularly on decision-leaders within the dog world and are open to all stakeholder groups in canine health. It was refreshing to see the spectrum of players interacting openly and with little apparent prejudice during the most recent meeting at the 3rd IDHW. With 140 participants from 23 countries, truly international views on canine health were shared and discussed. The value of this internationality was evident as groups explored the effects of differing national cultures, regulations and organizational structures on canine health programmes and prospects. Given the widespread movement of dogs and breeding material, both legally and illegally, between countries that was reported by many participants, it is clear that canine health in any one country does not exist in isolation but must be considered of substantial relevance to other countries. Participant diversity was equally underlined by the range of professional, organizational and interest-group stakeholders that attended. Organizations included kennel clubs/registration bodies, veterinary medical associations, welfare organizations, animal insurance, academia, regulators, media and foundations. The specific roles encompassed geneticists, veterinarians and epidemiologists, breeders, owners and dog-health campaigners, researchers, educators, data analysts, behavioral specialists and show judges. Many individuals have more than one affiliation and therefore carry out more than one role in relation to dogs. This eclectic mix of organizations and individuals promoted very healthy discussions and novel outcomes and actions. Exposure to opinion that was previously external to many groups was found to trigger original thoughts and solutions as well as building more cross-functional teams than those that normally tend to be assembled for canine health activities.

Despite the diversity of participants, two groups in particular could be encouraged to have greater input at future meetings. Although there were some individuals from each, these groups included the government and the media. First, although governments in most countries are well aware of, and often even involved in, the debate on canine health [75–77], their further engagement as collaborators towards effective solutions would be welcome. Governmental representatives could attend the 4th International Dog Health Workshop (4th IDHW) and, e.g. explain the challenges that regulators face in prioritizing effective canine welfare from a legal and political perspective. The power of media as an agent for both positive as well as negative change on the public psyche in relation to dogs is immense. The media can impact awareness of breed health and health-testing, basic canine health knowledge and trends towards breed popularity phenomena [5, 6]. Greater engagement by representatives from the media at future IDHW meetings could offer an effective route towards positive change in public opinion and behaviors.

### Next meeting

Each of the three previous IDHW meetings have occurred at locations that are easily accessible which has allowed delegates from all around the world to participate in these intensive meetings. In keeping with this, the 4th IDHW will be held in the UK from Thursday May 30th to Saturday June 1st, 2019 and will be organized by the UK Kennel Club. At this next workshop, the progress of the range of action plans specified within each theme at the 3rd IDHW will be presented and reviewed. It is anticipated that some of these actions may be completed and that the outcomes can be evaluated. For other actions that are still underway, the meeting will offer an opportunity to review progress and gather fresh input for potential acceleration. For actions that have yet to start or that have been deleted, the reasons for these results can be explored with a view to learning from failure as well as also searching for any opportunities to reset these goals. In addition, the 4th IDHW will allow the exploration of novel themes and the introduction of new delegates to the current worldwide collegiate from previous meetings. Efforts made to improve canine health must never stand still because new methods, knowledge and perspectives are constantly coming available and there are always fresh opportunities for progress.

## Conclusions

All three International Dog Health Workshop meetings have confirmed the benefits from inclusion of a diverse range of stakeholders who all play relevant and collaborative parts to improve future canine health. The 3rd IDHW expanded the emphasis on sustainable and measurable actions and outcomes, as well as information-sharing, discussion and networking. Participants were encouraged to share not only their expertise but also to update others on their current areas of work while holding open minds to new collaborations. So far, it appears that the workshop has been successful in terms of open sharing of information and tools, increasing connectivity and prioritization of main needs in canine health improvement. A number of exciting actions have also been agreed. The 4th IDHW will determine if these actions have been realized and whether meaningful improvements in canine health and welfare have been achieved.

## Abbreviations

1st IDHW: 1st International Dog Health Workshop 2012
2nd IDHW: 2nd International Dog Health Workshop 2015
3rd IDHW: 3rd International Dog Health Workshop 2017
4th IDHW: 4th International Dog Health Workshop 2019
AMR: Antimicrobial resistance
BOAS: Brachycephalic obstructive airway syndrome
CGE: Canine Genetics and Epidemiology
DALY: Disability adjusted life year
EBV: Estimated breeding values
FCI: Fédération Cynologique Internationale
GTP: Genetic test provider
IPFD: International Partnership for Dogs
KC: Kennel Club
QALY: Quality adjusted life year
QM: Quality measure
SMPH: Summary measures of population health
VDH: German Kennel Club
